# ALLEVIATING LONELINESS AT 100: IS RELIGION A SOLUTION?

**DOI:** 10.1093/geroni/igz038.149

**Published:** 2019-11-08

**Authors:** Alex J Bishop, Kevin Randall

**Affiliations:** 1 Oklahoma State University, Stillwater, Oklahoma, United States; 2 Sam Houston State University, Huntsville, Texas, United States

## Abstract

Data from N = 154 centenarians residing in Oklahoma were assessed using the Duke University Religious Index (DUREL). Items assessing religious salience (α=.76) were employed to create a binary measure of high (N=56 or 36.4%; M= 29.77, SD=4.65) and low (N=49 or 31.8%; M=25.10, SD=6.58) religious salience (RS). A series of ANCOVA analyses were then conducted controlling for education, race, self-reported health, and self-care capacity relative to the binary outcome RS. Significant differences for both the corrected model and the pairwise comparisons using Bonferroni adjustment emerged in favor (p ≤.001) of the high RS group (M HI =29.60; M LO=25.29) for life satisfaction and social provisions (M HI =82.43; M LO=76.62). However, the RS group was also significantly higher (p =.004) in reported loneliness (M HI =34.56; M LO=31.63). Implications of the findings for reducing loneliness among centenarians reporting high religious engagement are further highlighted.

